# A New Generation of Functional Tagged Proteins for HIV Fluorescence Imaging

**DOI:** 10.3390/v13030386

**Published:** 2021-02-28

**Authors:** João I. Mamede, Joseph Griffin, Stéphanie Gambut, Thomas J. Hope

**Affiliations:** 1 Department of Microbial Pathogens and Immunity, Rush University Medical Center, Chicago, IL 60612, USA; Stephanie_Gambut@rush.edu; 2 Department of Cell and Developmental Biology, Northwestern University, Chicago, IL 60611, USA; joseph.griffin@northwestern.edu

**Keywords:** fluorescent HIV, integration competent tagged viruses, HIV early-steps

## Abstract

During the last decade, there was a marked increase in the development of tools and techniques to study the molecular mechanisms of the HIV replication cycle by using fluorescence microscopy. Researchers often apply the fusion of tags and fluorophores to viral proteins, surrogate proteins, or dyes to follow individual virus particles while they progress throughout infection. The inclusion of such fusion motifs or surrogates frequently disrupts viral infectivity or results in a change of the wild-type phenotype. Here, we detail the construction and functional characterization of two new constructs where we fused fluorescent proteins to the N-terminus of HIV-1 Integrase. In the first, IN is recruited into assembling particles via a codon optimized Gag to complement other viral constructs, while the second is fused to a Gag-Pol expression vector fully capable of integration. Our data shows that N-terminal tagged IN is functional for integration by both recovery of integration of catalytically inactive IN and by the successful infectivity of viruses carrying only labeled IN. These tools will be important to study the individual behavior of viral particles and associate such behavior to infectivity.

## 1. Introduction

Since the discovery of human immunodeficiency virus (HIV) [1,2], the study of its molecular mechanisms of infection has allowed for the development of methods that specifically target viral replication. This has resulted in effective antiretroviral therapies with great outcomes for the HIV pandemic.

In order to infect a cell, HIV must perform an elaborate dance with immune target cells to successfully complete integration into the cell’s DNA. It must escape the deleterious host cell defense machinery and at the same time correctly exploit critical host cellular factors required for infection. The recent scrutiny of the early steps of HIV infection has led to the discovery of many cellular proteins that are involved in infection. The viral capsid, formed by multiple CA subunits, has been shown to be essential to the steps of infection after fusion until integration. Determinants in CA drive microtubule trafficking, the early stages of reverse transcription, nuclear translocation, the selection of preferential sites of HIV integration, and modulate the level of innate sensing responses [3].

The critical role for CA in multiple steps of the early steps of the HIV life cycle is accepted by the HIV research community, yet the field still intensively debates how the virus coordinates the potential loss of core integrity and CA shedding, while CA plays a functional role in downstream events required for successful integration and infection. Two other subjects are also under debate: which host factors and pathways play supportive and obstructive roles, and whether these events are conserved across cell types and cellular compartments. As not all fused HIV particles are able to infect cells and given that there is a distribution of distinct particle fates that occur post-fusion, it becomes important to distinguish between the behavior of particles that are able to infect a cell and particles that do not complete the process of infection and are degraded instead by the cell as we previously proposed [4].

To study the different aspects of the early steps of HIV infection, many research groups developed tools to visualize individual viral particles. The first methods that were developed exploited constructs where eGFP was fused to HIV-1 Vpr, and when added in trans in combination with non-tagged Vpr from the viral constructs, reverse transcription capable viruses were successfully assembled [5]. Other methods followed, such as the labeling of Integrase (IN) or CA with tetra-cysteine motifs that can then be labeled with FlaSH/ReAsH labels [6,7,8]. Although these methods proved useful, either compromised viral capsid assemblies, Vpr loss prior to nuclear translocation, or photo-instability of the dyes led to the inability to identify all viral particles during the early steps of infection (i.e up to integration). More recently, multiple groups have exploited the capabilities of Vpr incorporation into virions by coding a Protease (PR) cleavage site that is followed by IN fused to fluorescent proteins (Vpr C-terminus) [9,10,11]. Derivatives fusing fluorescent proteins to either the C-terminus or N-terminus of IN were developed. This allowed the observation of viral complexes by tagged IN that revealed detectable and measurable levels of CA in the nucleus. 

By labeling IN or Vpr, that interact with the reverse transcrition complex, it was therefore possible to follow viral particles while studying other aspects of infection such as the disassembly of the viral capsid and nuclear translocation. These observations could be further informed by using fluid phase markers that report fusion and capsid integrity loss [4,12,13,14] and other surrogates reporting capsid integrity [9,15]. More recently, fully fused fluorophores to the N-terminus or C-terminus of CA have been shown to follow assembled fluorophores into viral complexes to study the early steps of infection [16,17].

**Figure 1:**
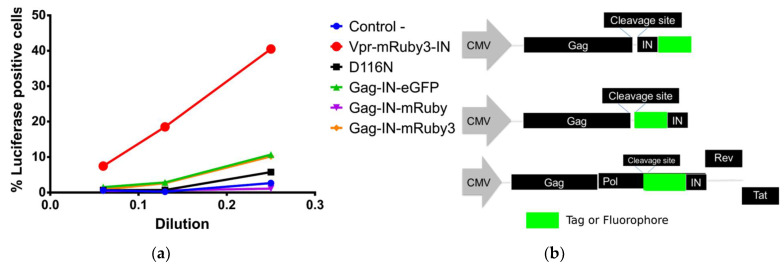
(**a**) C-terminal fusions to IN are integrase deficient while N-terminal IN fusions restore infectivity in HeLa cells with catalytically inactive IN from pNL4-3HIV-1 molecular clone. (**b**) Schematic organization of the fused constructs used in this study.

In parallel, we developed C-terminal fluorescent protein fusions to IN that are incorporated into the virions via Gag amplified from the HXB2 HIV-1 clone (Figure 1 Gag-IN-eGFP) [18]. The IN is preceded by Gag and its natural Protease (PR) cleavage site, resulting in the release of IN-eGFP from the precursor within particles after assembly and maturation [4,18,19]. The advantage of these constructs over Vpr-based incorporation is the fact that the most abundant protein that is assembled into an HIV-1 virion is Gag (>1500 copies per virion) [20,21]. This construct results in a one-to-one ratio between labeled IN and the trans incorporated Gag molecules from the transfected construct.

While C-terminal constructs have been successfully used in studies that followed viral particles from fusion to its presence in the nucleus, we have found that IN C-terminal fusion constructs are heavily impaired in its integration capabilities. We have observed this impairment in fully labelled complexes or in complexes with catalytically inactive IN (Figure 1a). However, other studies showed that the presence of this construct in the intasome does not negatively influence the functions of their wild type IN molecules [10]. Our experimental data confirms that N-terminal fusion constructs as reported by Dr. Ambrose’s laboratory [11] can restore intasome function when used in complement with the catalytically inactive IN-D116N mutant (Figure 1a).

A clear limitation of complementing with wild-type proteins (Vpr or IN) to individually detect HIV-1 particles, by adding tagged proteins in trans during viral production, is that it invariably leads to a subset of particles that might be unlabeled or labeled below the limit of detection by microscopy. This is particularly relevant when performing fluorescence live cell imaging as the fluorescence excitation must be kept at a minimum to prevent phototoxicity and photobleaching. Moreover, the presence of undetectable particles would confound the interpretation of results, as wild-type particles may more efficiently infect cells than the fusion fluorescent constructs being observed. Therefore, it is crucial to detect all viral particles challenging the cell when linking individual viral particle behavior to infectivity.

Here, we describe in detail the design, development, and validation of two “novel” viral constructs in which IN fused to diverse and commonly used fluorescent proteins was able to promote efficient integration of reverse transcribed DNA. These constructs are compatible with other constructs used in the field labeling the viral complex in different ways. By utilizing these constructs, we can produce fully labeled particle populations without any unlabeled wild-type particles being present. Therefore, these tools have the potential to be used in studies that seek to link the behavior of individual viral particles to successful or abortive infection as we previously proposed and pioneered [4].

## 2. Materials and Methods

### 2.1. Plasmids and Vector Cloning

#### 2.1.1. pCMV-optiGag-FluoIN

The aminoacid sequence from NL4-3 HIV-1 molecular clone was codon optimized in gag and was designed having the original NL4-3 coding sequence as input in Integrated DNA Technologies (IDT) website (Coralville, IA, USA) and codon optimized for *Homo sapiens*. The dsDNA fragments described in Supplementary File S1. were purchased from IDT (geneblocks).

pCMV-Gag-IN-eGFP [18] was digested with EcoRI + NotI and Hi-fi NEbuilder was used to construct the final vector by joining 3 fragments (pCMV vector, NL4-3 Codon Optimized *gag*, and fluorophore*IN* synthesized dsDNAs. Assemblies were transformed into electrocompetent Top10 bacteria (Thermo Fisher, Waltham, MA, USA) and spread onto kanamycin agar plates. Several colonies were screened, firstly by fragment digestion, and the whole modified sequence was verified by Sanger Sequencing. Maps in Supplementary File S2.

#### 2.1.2. psPAX2-FluoIN

psPAX2 was digested with EcoRV + AflII and the section between these two restriction sites were restored by fragments obtained by PCR (Q5 from NEB, Ipswich, MA, USA) using psPAX2 as template with the following oligos: EcoRV_PolFwd CAATGAGACACCAGGGATTAGAT; RTINRv TCCATCTAAAAATAGTACTTTCCTGAT as fragment 1; the pCMV-optiGag-FluoIN from several fluorophores were amplified with the oligos. RTINFwd ATCAGGAAAGTACTATTTTTAGATGGA; AflII_IN_Rv ACTGCCATTTGTACTGCTGTC as fragment 2; run on 0.8% agarose gels and purified with a gel extraction spin column (Sigma). The Vector and the 2 fragments were assembled using Hi-fi NEbuilder and cloned as detailed above for pCMV-optiGag-FluoIN using ampicillin agar plates. pl-EF1α-GFP-cppt was derived by the plKO.1-eGFP plasmid removing the U6 promoter meant to express shRNA with PspXI endonuclease restriction and restoring the cPPT with dsDNA oligos restoring the cPPT original sequence. The PGK promoter was replaced by EF1a that was amplified by PCR from pLentiCrisprV2 with the following oligos: Fwd GCTCGAGGAATTGGCTCCGGTGCCCGTCA; Rv GGATCCGTCCTGTGTTCTGGCGGCAAACC.

All final sequences were verified by Sanger Sequencing. Maps in Supplementary File S2.

### 2.2. Cells, Virus Production, and Quantification

HEK-293T (for virus production), 293-Affinofile and HeLa cells lines (measuring infections) were cultured in DMEM with 10% fetal bovine serum (Atlanta Biologicals), L-Glutamine, MEM-NEAA and antibiotics (Thermo Fisher Scientific).

**Figure 2:**
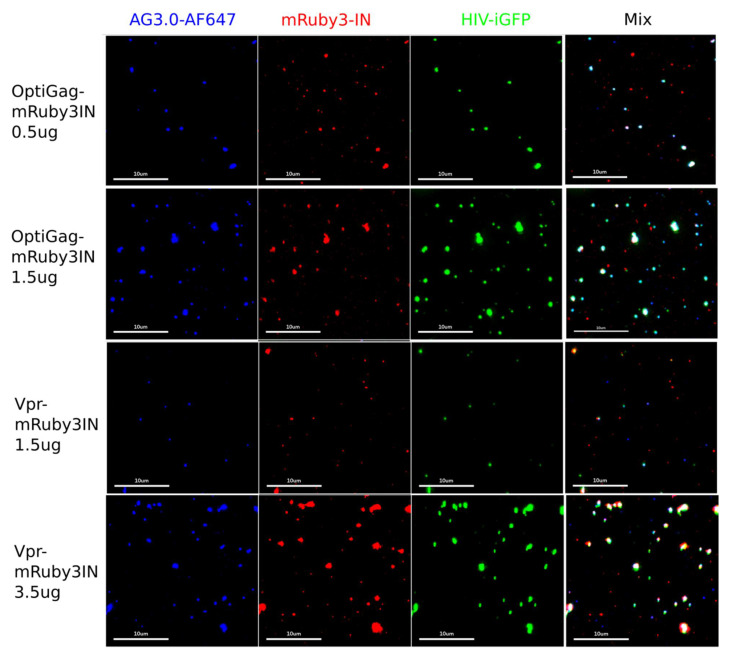
OptiGag-mRuby3IN and Vpr3-IN incorporate labeled IN into mature viral particles (detected with AG3.0 anti-CA antibody) by trans incorporation. The rows picture the imaging done with viruses produced by HEK-293T transfection with the shown different conditions, viruses were collected, and spun on glass. Each imaged channel is represented individually (Blue: anti CA AG3.0-AF647, Red: mRuby3IN, Green: HIV-iGFP) and mix represents the overlay of all the imaged channels. The percentage of dual labeled particles and intensity of each label varies depending on the transfection conditions such as the amount of transfected DNA (μg for each preparation are shown in the figure).

**Figure 3:**
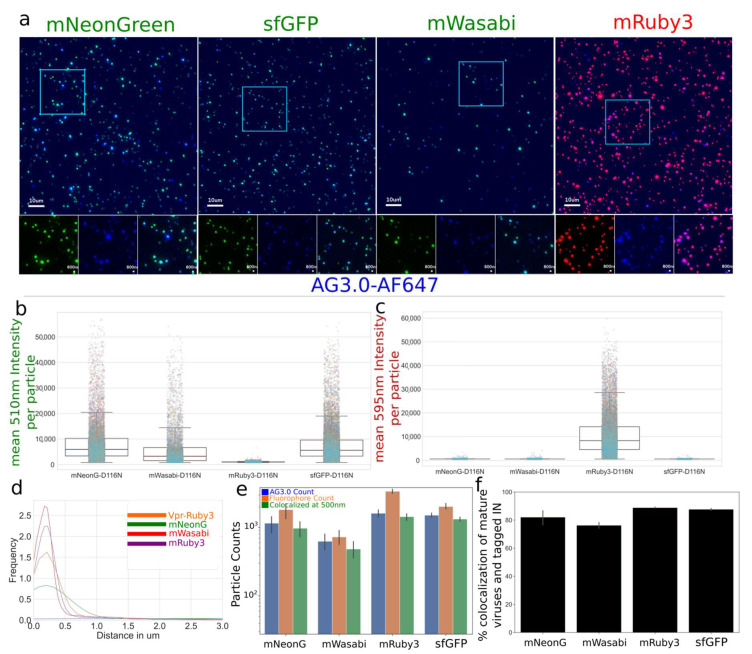
(**a**–**c**) Viruses spun on glass, fixed, and stained with AG3.0 antibody were produced with a non-fluorescent construct (pNL4-3-D116N-dEnv) that allows particle maturation by coding for HIV-1 Pol and high signal level in low excitation conditions. Green (mNeonGreen, sfGFP, or mWasabi), Red (mRuby3), Blue (mAB AG3.0 and anti-mouse-AF647) (**d**–**f**) As non-fluorescent particles are possible with *trans* complementation to pNL4-3 because the molecular clone does not code for any fluorescent protein, we quantified the frequency of labeled particles. We measured high levels of colocalization between mature particles, as identified by AG3.0 antibody, and the tagged IN, ranging from 75% to 90% colocalization. Colocalization was defined by centroids within 500nm of each other. Data represents one of the three repeated experiments with multiple technical repeats (>20) per experiment and thousands of viral particles analyzed (as shown in **b**).

For the optiGag experiments detailed in Figure 2 and Figure 3, HEK-293T cells were transfected with PEI with pLN4-3-D116N-dEnv, pCMV-VSV-G, and together with any of the pCMV-optiGag-FluoIN detailed in this manuscript together or pCMV-Vpr-R3IN. pLN4-3-D116N-dEnv and pCMV-Vpr-R3IN were kindly gifted by Dr. Zandrea Ambrose.

**Figure 4:**
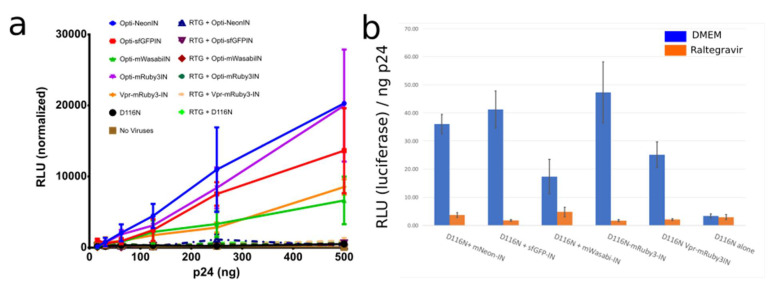
(**a**,**b**) Integration incompetent pNL4-3-D116N when produced with optiGag-Fluo supplemented in trans has integration restored to a higher level than Vpr-Ruby3-IN in HeLa cells. Raltegravir (RTG) was used as a control for IN inhibition of the labeled IN. Legend: Relative Light Units (RLU). Error Bars represent Standard Deviation of 3 independent experiments.

For the characterization of the psPAX2-FluoIN constructs in their fluorescence and infectivity measurements, we transfected pL-EF1a-eGFP-cPPT, pCMV-VSV-G, together with the respective psPAX2-FluoIN detailed in this manuscript (Figure 4).

A media change was performed 16 h post-transfection and viral supernatants were collected 36 h to 48 h post-transfection. The samples were subsequently filtered through a 0.45 μm PVDF filter (Millipore Sigma), aliquoted, and frozen at −80 °C. More than 3 different viral preparations of each shown viral construct were tested showing with similar results.

p24 quantification was done with R&D Systems HIV-1 Gag p24 Quantikine ELISA Kit according to the protocol provided by the vendor.

### 2.3. Virus on Glass Experiments

Virus on Glass (VOG) experiments were performed in viral preparations. Viral preps that incorporated fluorescent fusion constructs were spun between 1 h and 1 h 30 min at 15 °C at 2400 g in 96-well glass bottom plates previously treated with fibronectin. The media was then removed, and plates were washed with PBS. PIPES with 3.7% Formaldehyde (final concentration) was used to fix for five minutes, followed by 3 more washes with PBS. Viruses were permeabilized for 10 min in blocking buffer (10% Normal Donkey Serum, 0.01% NaN3, 0.1% Triton-X100) before staining at a 1:200 dilution for 1h with p24-AG3 antibody (NIH AIDS Reagent Program, Division of AIDS, NIAID, NIH). This antibody only stains mature HIV-1 capsids. The mAb was washed 3 times with PBS and stained with anti-mouse-AF647 antibodies (1:1000) for 45 min. Secondary antibodies were washed 3 times with PBS and imaged.

### 2.4. Immunofluorescence Imaging

Images were acquired with a Nikon wide-field Ti-e2 or DeltaVision Ultra widefield microscope (GE Life Sciences) equipped with an LED light sources and a EMCCD and sCMOS cameras using standard Chroma DAPI/GFP/TRITC/Cy5 polychroic and emission filters for DAPI/GFP/TRITC/Cy5. Images were deconvolved using flowdec (https://github.com/hammerlab/flowdec, accessed on 29 January 2021, release v1.1.0) or DeltaVision deconvolution package, Nominal magnification was 60× for all experiments. All images in the same panels were acquired with the same light, detection, and analysis conditions.

### 2.5. Particle Detection and Quantification

Virus on Glass images were deconvolved using flowdec with a theoretical PSF calculated with Fiji/ImageJ and Z max-intensity projected. Viral particles were detected using trackpy for each independent channel. In short, several attributes were collected from masks around the centroid calculated by trackpy for each independent channel. When calculating the % of co-localization, we used scipy kdtrees libraries to brute force calculate all the nearest-neighbors pairs to mature particles (as detected by AG3.0 positive puncta reporting mature virions) and particles to be co-localized when centroids were less than 0.500 µm apart.

### 2.6. Infection Analysis

**Figure 5:**
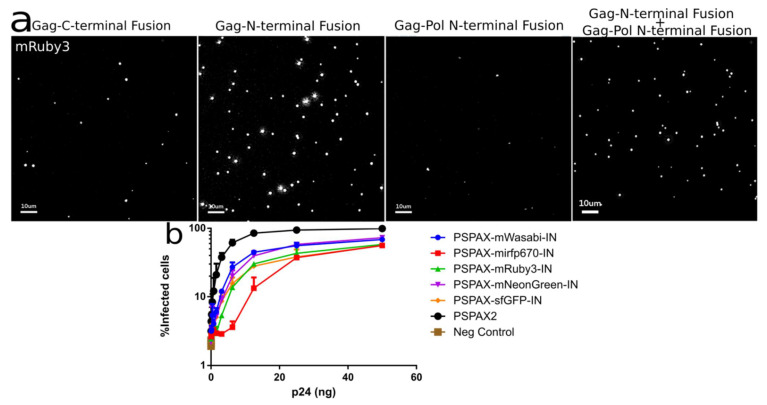
(**a**) Intensity of different IN labeled constructs by viral particle spinning on glass, Gag-INmRuby3, Gag-mRuby3IN and psPAX2-mRuby3IN (**b**) Fully labeled IN in Pol can integrate into 293 Affinofile cells at a ~70 to 80% level while compared with unlabeled wild-type IN. Error Bars represent standard deviation between two independent experiments. Kruskal–Wallis test shows a *p*-value < 0.01 between infection samples and non-infected cells (Neg. Control), all ranked Dunn’s multiple comparisons between all labelled infections do not show statistical significance to the alternative hypothesis that the labeled and unlabeled IN differ in % of infected cells.

HeLa cells were plated in 96-well plastic bottom dishes. Cells were challenged with the D116N + optiGag and polybrene for 48h while incubated at 37 ℃, 5% CO2. Two days after infection, the cells were lysed with ONE-Glo Luciferase Assay System, Promega, and luciferase luminosity was measured in a microplate reader (BioTek Cytation3). Experiments represented in Figure 2, Figure 3 and Figure 4 were performed in 3 independent experiments and Figure 5 in 2 independent experiments.

For psPAX2-FluoIN carrying viruses, the cells were infected with the same procedure, but after 48h cells were detached with 50 μL trypsin and fixed with 50 μL fix solution (4:1, PBS: 10% formaldehyde) and analysed with flow-cytometry for GFP expression. Singlet cells (FSC-H/FSC-W, SSC-H/SSH-W gating) for the diverse mentioned infections were analyzed by BD Fortessa FACS for the percentage of GFP signal using non-infected cells as control.

## 3. Results

Initially, we compared several constructs that were used in past publications [4,11,18,19] for their capacity to rescue HIV-1 integration activity when combined with a catalytically inactive IN (D116N) from a pNL4-3 firefly luciferase reporter vector. We produced pNL4-3-D116N-dEnv-Luc VSV-G pseudotyped virions, mixed with tagged IN where transfection was done in trans with Vpr or Gag constructs. We have found that C-terminal fusion to IN was not capable of rescuing the integration of the reverse transcribed DNA into the cell genome. This was measured by the production and luminescence of Firefly Luciferase coded by the pNL4-3-D116N construct. We have observed that N-terminal fusion of fluorophores to IN were capable of restoring infectivity as previously reported by others [11,22,23].

### 3.1. Development of Codon Optimized Gag-FluorophoreIN Constructs

After observing that N-terminal fusions to IN are capable of normal function as a fluorescently tagged protein, we designed new constructs aiming to incorporate high levels of fluorescently labeled IN into HIV-1 virions. We therefore produced pGag-Fluorophore-IN by placing coding sequences for a fluorescently tagged IN (Figure 1b) at the C-terminal end of Gag. The labeled IN is preceded by the natural protease cleavage site of IN so that IN is released from Gag after virus maturation. By doing so, we aimed to produce bright particles as Gag is the most abundant polyprotein in the virion [24]. We first constructed HXB2 and NL4-3 sequence-based plasmids, however whilst the HXB2 construct did present fluorescence HIV-1 particles (as pGAG-IN-Fluorophore), the NL4-3 based “Gag-FluorophoreIN” showed no fluorescence associated to viral particles (data not shown). This lack of mRNA transport is a well-known and established to be Rev dependent, where gag is known to be poorly expressed. This Rev dependance can be overcome by inserting regulatory elements such as the constitutive transport element (CTE). Moreover, a mechanistic connection between mRNA trafficking and correct assembly of Gag has been shown [25,26,27,28,29].

Taking into account the Rev dependency to mRNA export with wild-type sequences from the nucleus, we designed a fully synthetic construct where the coding NL4-3 Gag sequence was fully codon optimized for *Homo sapiens* (Sequences in Supplementary File S1). By conjugating the particle production between this construct and distinct pNL4-3 based constructs (pNL4-3dEnv or pHIV-iGFPdEnv), where the latter incorporates eGFP inside and outside of the capsid [4,12,30,31] we identified a high frequency of labeling (>85%) of the produced particles by spinning them onto glass, fixed and imaged by immunofluorescence with AG3.0 monoclonal antibody that only labels mature HIV-1 particles (Figure 2 and Figure 3a–e). By combining the HIV-iGFP, which has eGFP coded in gag, to the codon optimized Gag with a fluorescently labeled IN with mRuby3, we ensure a sufficient number of wild-type Gag molecules that stabilize the HIV-iGFP production that is known to be not as effective as wild-type. At the same time, we can be sure that every viral particle is labeled with either or both fluorophores, therefore avoiding confounding non-fluorescent viral particles in the preparations.

#### 3.1.1. Characterization and Quantification of Labeled IN Incorporation into Viral Particles

We then carried out a comprehensive characterization of viral preparations with optiGag constructs coding for different fluorophores carrying N-terminal fusions to IN (Figure 3a–c). The viruses were prepared using a single labeling system with a pNL4-3dEnv backbone. We imaged and measured a high degree of labeling that colocalizes with mature virions (Figure 3a–d). The colocalization ranged from 70% to 90% even in a configuration where unlabeled particles are possible (Figure 3e,f). The signal was acquired with light input similar to the conditions used in 48 h long live-cell imaging experiments where we use extremely low illumination power to prevent phototoxicity and cell death. We have found that the mRuby3, sfGFP and mNeonGreen viral preparations present very bright particles.

#### 3.1.2. OptiGag-FluoIN Restores Infectivity of Catalytically Inactive D116N Integrase

Several IN fusion constructs incorporated into viruses with Vpr have been used and detailed in other studies to visualize the behavior of individual viral particles as they proceed through infection [4,9,10,11,18,32]. A known limitation is that the usage of Vpr has a maximum amount of incorporation into viral particles. At the same time, that high amount can have an impact on infectivity. Also, the fusion of fluorophores to the C-terminal of HIV-1 IN is not capable of infection in a setting where all IN are labeled (Figure 1a,b). Thus, we used catalytically inactive IN with the mutation D116N and produced virions with distinct fluorophores fused to the N-terminus of IN. We challenged HeLa cells with the same viral input, measured by p24 ELISA, and observed that the labeled IN from our codon optimized Gag construct is able to restore DNA integration and Luciferase production to a high level even with a low amount of transfected optiGag DNA, whereas D116N viruses and all inoculations with raltegravir (RTG) presented no signal (Figure 4).

### 3.2. Development of Gag-Pol Constructs Carrying IN with N-Terminally Fused Fluorescent Proteins

OptiGag provides a method to ensure, at a genetic level, the production of viral preparations where all viral particles are fully fluorescently labeled when used together with a backbone that incorporates a fluorophore, such as HIV-iGFP (Figure 2). However, it will not be able to produce fully labeled viral particles using a non-labeled viral backbone (e.g HIV-pNL4-3), or a third-generation vector system, with viral proteins and the packaged mRNA with reporter expression, are split into multiple plasmids [33]. We then cloned a GagPol packaging system where the fluorescent proteins were inserted in IN that is coded in *pol* (Figure 1b).

#### Characterization and Quantification of Fully Labeled Fluorescent IN Labeled Viral Particles that Are Infectious

To produce our fully IN labeled viruses, we transfected 293T cells with G protein from VSV, controls or labeled GagPol, and a packaging vector expressing eGFP after integration. As expected, from the 1:20 Pol to Gag ratio [24] in the virions, the fluorescence signal was lower compared to the optiGag system detailed above and the C-terminal HXB2 system from other studies (Figure 5a) [4,18,19]. While the intensity of virion fluorescence was lower than the optiGag system, we ensure production of fully labeled particles at a genetic level as all IN are labeled. The infection, as determined by flow cytometry measurement of eGFP expression, shows a robust infectivity percentage that ranges from ~60% (sfGFP IN) to 73% (mNeonG IN) compared to non-labeled particles (Figure 5b). To overcome the lower fluorescence signal intensity by Pol that is fully labeled in *cis* with IN, we also transfected optiGag-NtermIN in trans, thus producing fully labeled particles with a high intensity of IN signal (Figure 5a).

## 4. Discussion

In recent years, there has been an effort by many research groups to develop methods that label individual particles to study different steps of the early phase of viral infection using different microscopy approaches. While these new methods have resulted in significant contributions to learning more about the HIV-1 life cycle, it has also led to confusion because the different systems generate very different outcomes. Thus, the field still intensively debates how the virus coordinates uncoating with these multiple steps of infection, how the viruses interact with the cell cytoskeleton, nuclear envelope, and nuclear contents, and which host factors and pathways play supportive or obstructive roles to infection. It is not possible to determine if these different systems reveal alternative functional pathways or if some of the labeling approaches are perturbing the system in a way that alter normal viral kinetics and dynamics leading to a distinct outcome of questionable biological relevance. For the field to move forward we need to establish a series of tests to characterize and compare the different labeled virion systems. 

Not all HIV-1 particles associating with a target cell are able to (i) fuse into the cytoplasm, (ii) reverse transcribe, (iii) translocate the nuclear pore, (iv) integrate its DNA into the host cell genome, and ultimately (v) produce infectious particles [34,35]. Given this stochastic nature of HIV-1 infection, where there is a distribution of distinct particle fates that occur post-fusion, it becomes important to distinguish between the behavior of particles that are able to infect a cell and particles that are ineffectual and degraded by the cell. Previously, we developed a live-cell imaging method that allows for differentiating between non-infectious and infectious HIV behavior by challenging cells with less than one particle per cell [4].

A highly limiting aspect of labeling viral particles with a single label in trans*,* or with surrogates that identify viral particles, is that very often the HIV-1 backbone is not expressing the labeled protein. This results in a competition between wild-type and labeled proteins during viral assembly. This can yield significant amounts of wild-type particles, that will likely be more efficient in infection. This aspect of particle generation leads to the creation of unlabeled particles that cannot be observed by live cell imaging, introduce noise into the system, and could be responsible for skewing the data to indicate a greater number of infectious events that lead to an overestimation of infectious titer from the labeled particles. In contrast, labeled viruses could have a fitness handicap that would be hidden by the contaminating unlabeled wild-type virus. Moreover, several of these labeling methods were only partially characterized for their ability to recapitulate the molecular behavior of non-labeled wild-type HIV-1 particles. Such a characterization is often only performed in a particular step or a subset of aspects of the HIV-1 replication cycle that is relevant to a given study. A thorough characterization of all new labeling approaches needs to be extensively vetted.

In this study, we report the design and validation of new methods that genetically remove the possibility of unlabeled particles. Both the optiGag-FluoIN and psPAX2-FluoIN constructs allow for a high level of integration activity and capacity by the HIV-1 intasome. The former also allows for a high level of IN labeling into the particles. While psPAX2, genetically, will code and package similar levels of IN into the virion, the same does not happen to optiGag-FluoIN. It is therefore possible that the extra IN packed in a virion could influence the infectivity levels or mechanistic behavior of the virion. This would warrant further examination when applied in future studies to ascertain the possible effect in such stages of the viral replication cycle, such as trafficking and uncoating. 

When using new viral constructs to study the molecular aspects of HIV infection, they should be properly validated and compared to other methods that have been previously used. Some of these validation methods may include functional, drug add-ins and washouts, biochemical assays, and new approaches that evaluate the kinetics of nuclear import [36]. It is likely that the introduction of bulky fluorescent tags, which are often the same or higher in size than the viral proteins (i.e., CA, RT, IN), disrupts the correct functioning of the virus depending on the site of fusion. It is also possible that it might result in a blockage of the natural interactions between the viral proteins. Another aspect is that the fluorescent signal might not represent the wild-type viral behavior being studied and their correct incorporation into the virion. This becomes critical when mixing a high level of wild-type viral proteins with tagged fluorophores, as the fluorescent signal might not represent the natural behavior and functioning of viral structures, such as the intasome or capsids.

These HIV-1 IN constructs can be used to qualify and quantify the entirety of the early steps of HIV-1 infection by identifying viral complexes, while at the same time they complement labeled constructs (e.g., HIV-iGFP, HIV-gagGFP, S15/DiD/DiO/DiL labeling, others) that study other aspects of infection such as fusion and capsid shedding where such labels are lost before integration to the cell. In parallel, we developed a Gag-Pol construct that can produce fully labeled particles while retaining a high level of infectivity allowing for the imaging of IN containing viral complexes to study the entirety of the early steps of infection.

## Data Availability

All data and detection computing scripts will be shared upon request to J.I.M.

## Supplementary Materials

The following are available online at https://www.mdpi.com/1999-4915/13/3/386/s1, File S1: Synthetic genes used for optiGag-mRuby3IN. File S2: Plasmids Maps.
