# Supplementary material for: A New Generation of Functional Tagged Proteins for HIV Fluorescence Imaging
Source: Viruses. 2021 Feb 28;13(3):386. doi: 10.3390/v13030386 (PMC7997544; doi:10.3390/v13030386)

Supplementary Materials:

File S1

Synthetic genes used for optiGag-mRuby3IN

NL4-3 Codon Optimized Gag fragment:

ATCTCGAGCTCAAGCTTCGAATTCGCCACCATGGGTGCGCGAGCGTCCGTGCTGAGTGGCGGCGAACTCGAC
AAATGGGAAAAGATCCGGCTCAGACCTGGGGGAAAGAAACAATACAAGCTTAAACACATCGTTTGGGCCAG
CCGCGAGCTGGAGAGGTTTGCCGTTAATCCTGGGCTTCTGGAAACGAGTGAAGGTTGCAGGCAAATTCTCGG
ACAACTTCAGCCCTCTCTCCAAACTGGTTCTGAAGAACTCCGCAGTTTGTACAATACTATTGCGGTTCTTTATT
GTGTCCACCAACGCATAGACGTGAAAGATACTAAGGAGGCGCTGGACAAAATCGAAGAAGAGCAGAATAA
GAGCAAAAAAAAAGCCCAGCAGGCCGCGGCTGACACCGGCAACAATAGCCAGGTCAGTCAAAACTACCCC
ATTGTCCAGAATCTTCAAGGACAAATGGTACATCAAGCGATCTCTCCCCGAACATTGAATGCCTGGGTGAAG
GTCGTCGAAGAAAAAGCCTTTAGCCCAGAAGTCATTCCGATGTTTTCTGCACTTTCTGAGGGCGCAACGCCGC
AGGACCTGAACACTATGCTTAATACAGTAGGCGGACATCAAGCAGCAATGCAAATGCTGAAAGAGACCATT
AATGAGGAGGCTGCCGAGTGGGATCGGCTTCACCCTGTGCATGCAGGTCCGATTGCCCCGGGCCAGATGCGC
GAGCCAAGAGGATCAGATATCGCTGGAACAACTTCTACGTTGCAAGAGCAAATTGGTTGGATGACACATAAC
CCTCCCATTCCCGTGGGCGAGATTTACAAGAGGTGGATAATACTGGGTTTGAATAAAATTGTGAGAATGTACT
CACCAACCTCAATCTTGGACATAAGACAAGGTCCAAAGGAGCCCTTCAGAGACTACGTCGACAGGTTTTACA
AGACCCTCCGAGCTGAACAGGCTAGCCAGGAAGTCAAGAATTGGATGACCGAAACACTTCTTGTTCAAAATG
CAAATCCAGATTGTAAGACTATCCTGAAGGCTCTGGGACCTGGAGCCACGTTGGAGGAAATGATGACCGCTT
GTCAGGGAGTAGGCGGGCCCGGTCACAAAGCTCGGGTCCTGGCTGAAGCAATGAGCCAGGTCACCAACCCG
GCCACCATCATGATCCAAAAGGGGAATTTCCGAAATCAGCGAAAGACTGTAAAGTGTTTCAATTGTGGGAAA
GAGGGGCACATCGCAAAGAATTGTCGAGCTCCACGAAAAAAGGGGTGTTGGAAATGTGGAAAGGAAGGGC
ATCAAATGAAGGATTGCACGGAAAGGCAAGCTAATTTCTTGGGTAAAATTTGGCCCTCTCATAAGGGACGAC
CAGGGAACTTTCTCCAATCCAGGCCTGAGCCAACAGCCCCGCCCGAGGAATCTTTCAGGTTCGGCGAGGAGA
CGACTACACCCAGCCAGAAACAGGAACCAATTGACAAGGAGTTGTATCCTCTGGCATCCCTTCGGAGTTTGT
TCGGTTCAGACCCCTCGTCACAAGG

mRuby3IN fragment

GACCCCTCGTCACAAGGGGAAGTAGATAAATTAGGTGCTGGTATCCGTAAAGTACTATTTTTAGATGGAATA
GATATGGTGTCTAAGGGCGAAGAGCTGATCAAGGAAAATATGCGTATGAAGGTGGTCATGGAAGGTTCGGTC
AACGGCCACCAATTCAAATGCACAGGTGAAGGAGAAGGCAGACCGTACGAGGGAGTGCAAACCATGAGGA
TCAAAGTCATCGAGGGAGGACCCCTGCCATTTGCCTTTGACATTCTTGCCACGTCGTTCATGTATGGCAGCCG
TACTTTTATCAAGTACCCGGCCGACATCCCTGATTTCTTTAAACAGTCCTTTCCTGAGGGTTTTACTTGGGAAA
GAGTTACGAGATACGAAGATGGTGGAGTCGTCACCGTCACGCAGGACACCAGCCTTGAGGATGGCGAGCTC
GTCTACAACGTCAAGGTCAGAGGGGTAAACTTTCCCTCCAATGGTCCCGTGATGCAGAAGAAGACCAAGGGT
TGGGAGCCTAATACAGAGATGATGTATCCAGCAGATGGTGGTCTGAGAGGATACACTGACATCGCACTGAAA
GTTGATGGTGGTGGCCATCTGCACTGCAACTTCGTGACAACTTACAGGTCAAAAAAGACCGTCGGGAACATC
AAGATGCCCGGTGTCCATGCCGTTGATCACCGCCTGGAAAGGATCGAGGAGAGTGACAATGAAACCTACGT
AGTGCAACGCGAAGTGGCAGTTGCCAAATACAGCAACCTTGGTGGTGGCATGGACGAGCTGTACAAGGGAT
CCGCTGGCTCCGCTGCTGGTTCTGGCGAATTCTTTTTAGATGGAATAGATAAGGCCCAAGAAGAACATGAGA
AATATCACAGTAATTGGAGAGCAATGGCTAGTGATTTTAACCTACCACCTGTAGTAGCAAAAGAAATAGTAG
CCAGCTGTGATAAATGTCAGCTAAAAGGGGAAGCCATGCATGGACAAGTAGACTGTAGCCCAGGAATATGG
CAGCTAGATTGTACACATTTAGAAGGAAAAGTTATCTTGGTAGCAGTTCATGTAGCCAGTGGATATATAGAA
GCAGAAGTAATTCCAGCAGAGACAGGGCAAGAAACAGCATACTTCCTCTTAAAATTAGCAGGAAGATGGCC
AGTAAAAACAGTACATACAGACAATGGCAGCAATTTCACCAGTACTACAGTTAAGGCCGCCTGTTGGTGGGC
GGGGATCAAGCAGGAATTTGGCATTCCCTACAATCCCCAAAGTCAAGGAGTAATAGAATCTATGAATAAAG
AATTAAAGAAAATTATAGGACAGGTAAGAGATCAGGCTGAACATCTTAAGACAGCAGTACAAATGGCAGTA
TTCATCCACAATTTTAAAAGAAAAGGGGGGATTGGGGGGTACAGTGCAGGGGAAAGAATAGTAGACATAAT
AGCAACAGACATACAAACTAAAGAATTACAAAAACAAATTACAAAAATTCAAAATTTTCGGGTTTATTACA
GGGACAGCAGAGATCCAGTTTGGAAAGGACCAGCAAAGCTCCTCTGGAAAGGTGAAGGGGCAGTAGTAATA

CAAGATAATAGTGACATAAAAGTAGTGCCAAGAAGAAAAGCAAAGATCATCAGGGATTATGGAAAACAGA
TGGCAGGTGATGATTGTGTGGCAAGTAGACAGGATGAGGATTAAGCGGCCGCGACTCTAGATCATAATCAG

sfGFP-IN fragment

GGTGCTGGTATCCGTAAAGTACTATTTTTAGATGGAATAGATATGAGCAAAGGAGAAGAACTTTTCACTGGA
GTTGTCCCAATTCTTGTTGAATTAGATGGTGATGTTAATGGGCACAAATTTTCTGTCCGTGGAGAGGGTGAAG
GTGATGCTACAAACGGAAAACTCACCCTTAAATTTATTTGCACTACTGGAAAACTACCTGTTCCATGGCCAAC
ACTTGTCACTACTCTGACCTATGGTGTTCAATGCTTTTCCCGTTATCCGGATCACATGAAACGGCATGACTTTT
TCAAGAGTGCCATGCCCGAAGGTTATGTACAGGAACGCACTATATCTTTCAAAGATGACGGGACCTACAAGA
CGCGTGCTGAAGTCAAGTTTGAAGGTGATACCCTTGTTAATCGTATCGAGTTAAAAGGTATTGATTTTAAAGA
AGATGGAAACATTCTCGGACACAAACTCGAGTACAACTTTAACTCACACAATGTATACATCACGGCAGACAA
ACAAAAGAATGGAATCAAAGCTAACTTCAAAATTCGCCACAACGTTGAAGATGGTTCCGTTCAACTAGCAGA
CCATTATCAACAAAATACTCCAATTGGCGATGGCCCTGTCCTTTTACCAGACAACCATTACCTGTCGACACAA
TCTGTCCTTTCGAAAGATCCCAACGAAAAGCGTGACCACATGGTCCTTCTTGAGTTTGTAACTGCTGCTGGGA
TTACACATGGCATGGATGAGCTCTACAAAAAGGGATCCGCTGGCTCCGCTGCTGGTTCTGGCGAATTCTTTTT
AGATGGAATAGATAAGGCCCAAGAAGAACATGAGAAATATCACAGTAATTGGAGAGCAATGGCTAGTGATT
TTAACCTACCACCTGTAGTAGCAAAAGAAATAGTAGCCAGCTGTGATAAATGTCAGCTAAAAGGGGAAGCC
ATGCATGGACAAGTAGACTGTAGCCCAGGAATATGGCAGCTAGATTGTACACATTTAGAAGGAAAAGTTATC
TTGGTAGCAGTTCATGTAGCCAGTGGATATATAGAAGCAGAAGTAATTCCAGCAGAGACAGGGCAAGAAAC
AGCATACTTCCTCTTAAAATTAGCAGGAAGATGGCCAGTAAAAACAGTACATACAGACAATGGCAGCAATTT
CACCAGTACTACAGTTAAGGCCGCC

mWasabi-IN fragment

GGTGCTGGTATCCGTAAAGTACTATTTTTAGATGGAATAGATATGGTGAGCAAGGGCGAGGAGACCACAATG
GGCGTAATCAAGCCCGACATGAAGATCAAGCTGAAGATGGAGGGCAACGTGAATGGCCACGCCTTCGTGAT
CGAGGGCGAGGGCGAGGGCAAGCCCTACGACGGCACCAACACCATCAACCTGGAGGTGAAGGAGGGAGCC
CCCCTGCCCTTCTCCTACGACATTCTGACCACCGCGTTCAGTTACGGCAACAGGGCCTTCACCAAGTACCCCG
ACGACATCCCCAACTACTTCAAGCAGTCCTTCCCCGAGGGCTACTCTTGGGAGCGCACCATGACCTTCGAGG
ACAAGGGCATCGTGAAGGTGAAGTCCGACATCTCCATGGAGGAGGACTCCTTCATCTACGAGATACACCTCA
AGGGCGAGAACTTCCCCCCCAACGGCCCCGTGATGCAGAAGGAGACCACCGGCTGGGACGCCTCCACCGAG
AGGATGTACGTGCGCGACGGCGTGCTGAAGGGCGACGTCAAGATGAAGCTGCTGCTGGAGGGCGGCGGCCA
CCACCGCGTTGACTTCAAGACCATCTACAGGGCCAAGAAGGCGGTGAAGCTGCCCGACTATCACTTTGTGGA
CCACCGCATCGAGATCCTGAACCACGACAAGGACTACAACAAGGTGACCGTTTACGAGATCGCCGTGGCCC
GCAACTCCACCGACGGCATGGACGAGCTGTACAAGAAGGGATCCGCTGGCTCCGCTGCTGGTTCTGGCGAAT
TCTTTTTAGATGGAATAGATAAGGCCCAAGAAGAACATGAGAAATATCACAGTAATTGGAGAGCAATGGCTA
GTGATTTTAACCTACCACCTGTAGTAGCAAAAGAAATAGTAGCCAGCTGTGATAAATGTCAGCTAAAAGGGG
AAGCCATGCATGGACAAGTAGACTGTAGCCCAGGAATATGGCAGCTAGATTGTACACATTTAGAAGGAAAA
GTTATCTTGGTAGCAGTTCATGTAGCCAGTGGATATATAGAAGCAGAAGTAATTCCAGCAGAGACAGGGCAA
GAAACAGCATACTTCCTCTTAAAATTAGCAGGAAGATGGCCAGTAAAAACAGTACATACAGACAATGGCAG
CAATTTCACCAGTACTACAGTTAAGGCCGCC

mNeonGreen-IN fragment
GGTGCTGGTATCCGTAAAGTACTATTTTTAGATGGAATAGATATGGTGAGCAAGGGCGAGGAGGATAACATG
GCCTCTCTCCCAGCGACACATGAGTTACACATCTTTGGCTCCATCAACGGTGTGGACTTTGACATGGTGGGTC
AGGGCACCGGCAATCCAAATGATGGTTATGAGGAGTTAAACCTGAAGTCCACCAAGGGTGACCTCCAGTTCT
CCCCCTGGATTCTGGTCCCTCATATCGGGTATGGCTTCCATCAGTACCTGCCCTACCCTGACGGGATGTCGCCT
TTCCAGGCCGCCATGGTAGATGGCTCCGGATACCAAGTCCATCGCACAATGCAGTTTGAAGATGGTGCCTCC
CTTACTGTTAACTACCGCTACACCTACGAGGGAAGCCACATCAAAGGAGAGGCCCAGGTGAAGGGGACTGG
TTTCCCTGCTGACGGTCCTGTGATGACCAACTCGCTGACCGCTGCGGACTGGTGCAGGTCGAAGAAGACTTAC
CCCAACGACAAAACCATCATCAGTACCTTTAAGTGGAGTTACACCACTGGAAATGGCAAGCGCTACCGGAG
CACTGCGCGGACCACCTACACCTTTGCCAAGCCAATGGCGGCTAACTATCTGAAGAACCAGCCGATGTACGT
GTTCCGTAAGACGGAGCTCAAGCACTCCAAGACCGAGCTCAACTTCAAGGAGTGGCAAAAGGCCTTTACCG
ATGTGATGGGCATGGACGAGCTGTACAAGAAGGGATCCGCTGGCTCCGCTGCTGGTTCTGGCGAATTCTTTTT
AGATGGAATAGATAAGGCCCAAGAAGAACATGAGAAATATCACAGTAATTGGAGAGCAATGGCTAGTGATT

TTAACCTACCACCTGTAGTAGCAAAAGAAATAGTAGCCAGCTGTGATAAATGTCAGCTAAAAGGGGAAGCC
ATGCATGGACAAGTAGACTGTAGCCCAGGAATATGGCAGCTAGATTGTACACATTTAGAAGGAAAAGTTATC
TTGGTAGCAGTTCATGTAGCCAGTGGATATATAGAAGCAGAAGTAATTCCAGCAGAGACAGGGCAAGAAAC
AGCATACTTCCTCTTAAAATTAGCAGGAAGATGGCCAGTAAAAACAGTACATACAGACAATGGCAGCAATTT
CACCAGTACTACAGTTAAGGCCGCC

File S2

Plasmids Maps:
pOptiGag-mRuby3IN (all other optiGag-FluoIN plasmids with other fluorescent proteins share the same construct):

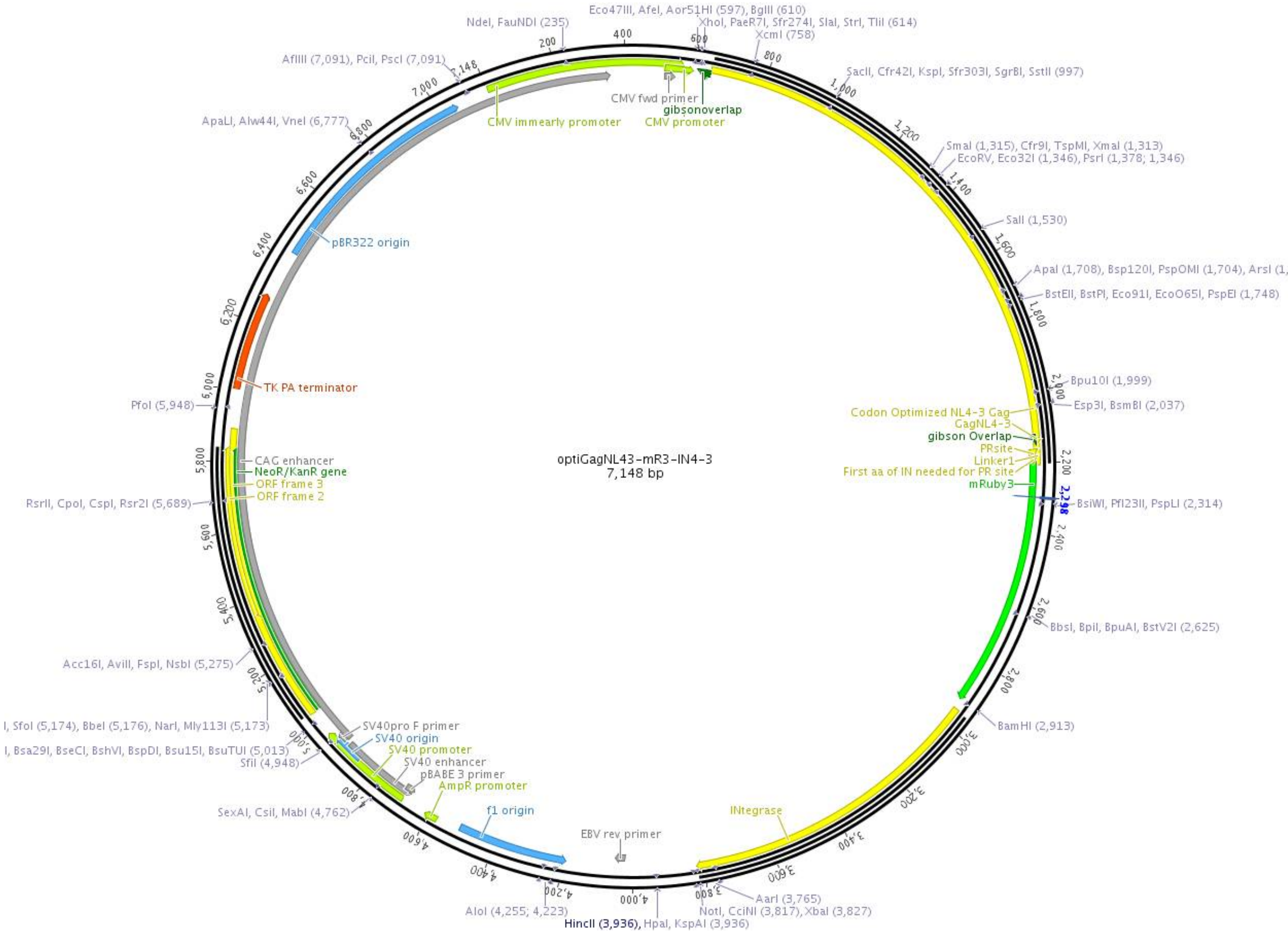

psPAX2-sfGFPIN (all other psPAX2-FluoIN plasmids with other fluorescent proteins share the same construct)

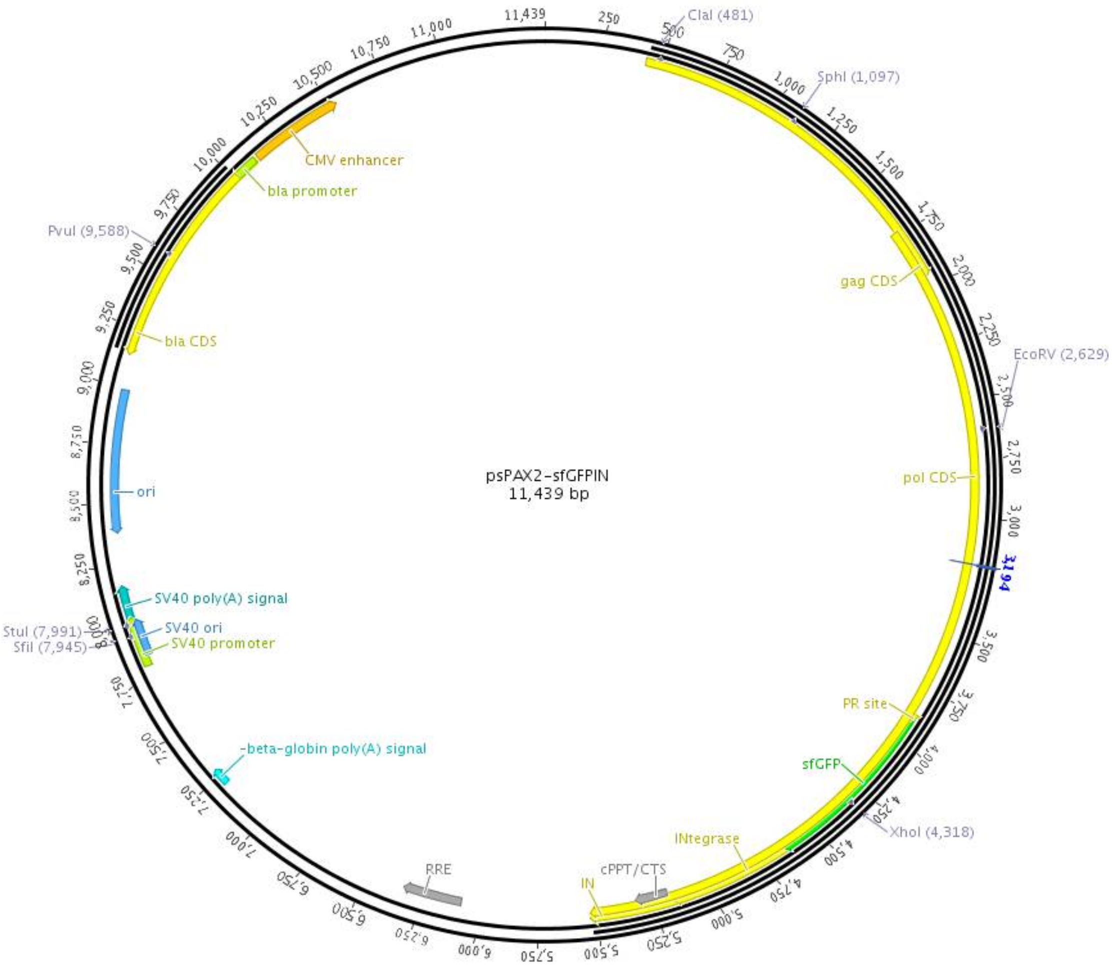

Supplement: Supplementary file 1 [file viruses-13-00386-s001.pdf]
